# Microclimatic temperatures at Danish cattle farms, 2000–2016: quantifying the temporal and spatial variation in the transmission potential of Schmallenberg virus

**DOI:** 10.1186/s13071-018-2709-8

**Published:** 2018-03-05

**Authors:** Najmul Haider, Ana Carolina Cuellar, Lene Jung Kjær, Jens Havskov Sørensen, Rene Bødker

**Affiliations:** 1 National Veterinary Institute, Technical University of Denmark, Copenhagen, Denmark; 2 Research and Development Department, Danish Meteorological Institute, Copenhagen, Denmark

**Keywords:** Schmallenberg virus, Microclimatic temperatures, EIP, Spatio-temporal modeling, Denmark, Vector-borne diseases, Transmission, *Culicoides* spp., Resting sites, Cattle farm

## Abstract

**Background:**

Microclimatic temperatures provide better estimates of vector-borne disease transmission parameters than standard meteorological temperatures, as the microclimate represent the actual temperatures to which the vectors are exposed. The objectives of this study were to quantify farm-level geographic variations and temporal patterns in the extrinsic incubation period (EIP) of Schmallenberg virus transmitted by *Culicoides* in Denmark through generation of microclimatic temperatures surrounding all Danish cattle farms.

**Methods:**

We calculated the hourly microclimatic temperatures at potential vector-resting sites within a 500 m radius of 22,004 Danish cattle farms for the months April to November from 2000 to 2016. We then modeled the daily EIP of Schmallenberg virus at each farm, assuming vectors choose resting sites either randomly or based on temperatures (warmest or coolest available) every hour. The results of the model output are presented as 17-year averages.

**Results:**

The difference between the warmest and coolest microhabitats at the same farm was on average 3.7 °C (5th and 95th percentiles: 1.0 °C to 7.8 °C). The mean EIP of Schmallenberg virus (5th and 95th percentiles) for all cattle farms during spring, summer, and autumn was: 23 (18–33), 14 (12–18) and 51 (48–55) days, respectively, assuming *Culicoides* select resting sites randomly. These estimated EIP values were considerably shorter than those estimated using standard meteorological temperatures obtained from a numerical weather prediction model for the same periods: 43 (39–52), 21 (17–24) and 57 (55–58) days, respectively. When assuming that vectors actively select the coolest resting sites at a farm, the EIP was 2.3 (range: 1.1 to 4.1) times longer compared to that of the warmest sites at the same farm.

**Conclusions:**

We estimated a wide range of EIP in different microclimatic habitats surrounding Danish cattle farms, stressing the importance of identifying the specific resting sites of vectors when modeling vector-borne disease transmission. We found a large variation in the EIP among different farms, suggesting disease transmission may vary substantially between regions, even within a small country. Our findings could be useful for designing risk-based surveillance, and in the control and prevention of emerging and re-emerging vector-borne diseases.

## Background

Schmallenberg is an emerging *Culicoides*-borne disease affecting cattle, sheep and goats, and is characterized by pyrexia, reduced milk production, abortion and congenital malformations in the offspring of infected animals [1–3]. Schmallenberg virus was detected in Germany for the first time in November 2011 [1] after which the virus spread to most of the countries in central and northern Europe, including Denmark in 2012 [4]. The virus development rate in insects, known as the extrinsic incubation period (EIP), is the time interval between ingestion of an infected blood meal and the ability to transmit the virus to a new host [5]. The EIP is highly dependent on the temperature surrounding the biting midges [6–8], which is called the microclimatic temperature [9]. The microclimatic temperature of a small geographic area is highly influenced by the presence and intensity of solar radiation, the level of humidity, the speed and direction of the wind, the topography, aspect and local precipitation [9, 10]. These factors are affected by vegetation and land cover, which therefore play an important role in determining the microclimatic temperatures in the available resting sites surrounding a cattle farm [10].

Vector-borne disease transmission models commonly use the temperature recorded by meteorological weather stations [7, 11]. Meteorological temperatures are recorded by weather stations according to the standards set up by the World Meteorological Organization (WMO) [12]. The weather stations are set up at very specific heights all over the world (generally 2 m above the ground), using a specific (white) colored box and placed in a way to protect the thermometer sensor from direct sunlight. As the WMO instructed, a WMO weather station site should be representative of a large area (i.e. 100–1000 km^2^) [12]. The area a weather station represents might have a number of different microclimatic habitats and therefore, the standard meteorological temperature does not fully represent all the different climatic condition of insect microhabitats [10, 13, 14]. When these meteorological temperatures are used in vector-borne disease transmission models, the models ignore the real temperature in the microhabitats the insects are actually exposed to [10, 13, 15]. These weather stations may be located as far as 50–100 km from the cattle farms in question and, more importantly, the meteorological temperature recorded by the weather station will only represent one of many potential microclimates of the area. The use of microclimatic temperature in disease modeling is hindered by the lack of data from microclimatic environments [10].

Previous studies have shown that many habitats have warmer microclimatic temperatures than the standard meteorological temperatures [10, 14, 15]. Even when the average daily microclimatic temperature is similar to the average meteorological temperature, the microclimatic temperature is more extreme being relatively warmer during the day and cooler during the night [10, 15]. Virus development in insect vectors is highly dependent on temperature [6, 7], but the relationship is not linear and often shows a threshold temperature, below which virus development is not possible [7, 8, 16]. Therefore, the higher daytime microclimatic temperatures result in average virus development times that are often much shorter than development times at meteorological temperatures [10].

Farm-level microclimatic temperature is not available from registers in Denmark or potentially the rest of Europe. Furthermore, vector-borne disease transmission is rarely assessed at the individual farm level, although a recent study predicted the potential of between-farm transmission of Schmallenberg virus in the UK [17]. The model showed that Schmallenberg virus can infect more farms and spread considerably further than bluetongue virus in the same time frame.

The resting site of an insect refers to the places where the insect rests after taking a blood meal [18, 19]. *Culicoides* spend almost 90% of their life time resting during which they develop oocysts to the appropriate stage for acquiring a blood meal, digestion of the blood meal and developing eggs [18].

The resting sites of biting midges in Denmark are largely unknown, but different species of biting midges may prefer different types and heights of vegetation [20]. A study in the Netherlands found *Culicoides* spp. in wetlands, peat bogs, riverine areas and livestock farms, but higher numbers of biting midges were recorded in wetland areas and peat bogs [15]. Carpenter et al. [21] found *Culicoides impunctatus* in very high numbers on European white birch (*Betula pubescens*), a deciduous tree native to northern Europe. Carpenter [22] reported adult *Culicoides* spp. resting on ground litter and on the underside of foliage. He also found adult *Culicoides* spp. equally distributed at 2.2, 7.0 and 10.7 m above ground [22]. Biting midges seek favorable microhabitats, and their choice is driven by temperature and humidity [22, 23]*.* However, little is known about their resting behavior at low Scandinavian temperatures during spring and autumn, when their abundance can be high [24]. Biting midges are able to move between different resting sites on a farm in order to optimize the conditions. The distance to which they are willing to move to find a suitable resting habitat is not known. However, Myers [25] found that biting midges can move up to 800 m in The Bahamas. Bidlingmayer [26] found *C. impunctatus* in Scotland, dispersed around 75 m from the original site of detection. Kirkeby et al. [27] recaptured marked biting midges at a distance of 1.75 km from their release point in Denmark.

The importance of microclimates has been emphasized in previous studies. For example, studies of highland malaria showed that anophelines may rest at warmer indoor temperatures [16, 28, 29], but were also found to be important in sea-level urban settings in Chennai, India where microclimatic temperature contributed to a shortened EIP for both vivax and falciparum malarial parasites [14]. Other studies have shown that air temperature has a substantial impact on malaria transmission across Africa [30]. Microclimatic temperatures provide significantly different estimates of vector-borne disease transmission parameters compared to meteorological temperatures [10]. Microclimatic temperatures allow for a faster pathogen development in biting midges (Schmallenberg and bluetongue virus) and mosquitoes (malaria, dengue, Dirofilaria and West Nile virus), more rapid digestion of blood meals, and a longer transmission season compared to meteorological temperatures [10].

Cattle farms may have one temperature recorded/modeled by the national meteorological service for any particular time period, but the farms are often surrounded by a number of microhabitats (vegetation) with potentially different microclimatic temperatures [10]. A previous study in Denmark showed that four microclimatic habitats located within a 1 km radius included a wide variation in temperature, and the temperature of these habitats was different from the nearest Danish Meteorological Institute (DMI) weather station [10]. Furthermore, the microclimatic temperature varied in different microhabitats, in different seasons and in different altitude. For example, the dry meadow was in general warmer than the hedges, wet meadow and forest. During spring, the lower heights of the dry meadow were warmer than the upper and mid-height whereas, during summer and autumn, the temperature at the lower habitats became cooler. The variation in vegetation types in different seasons played a vital role in changing the microclimatic temperature of different habitats [10]. Therefore, it is important to understand the microclimatic temperature of insect habitats surrounding the cattle farm over time and space. In this study, we explored the temperature of potential resting sites for *Culicoides* spp. from existing microclimatic habitats at all Danish cattle farms over 17 consecutive transmission seasons. The objective of this study was to quantify the variation in the EIP of Schmallenberg virus among these farms, and to identify possible spatial and temporal patterns of the EIP using the generated microclimatic temperatures.

## Methods

We obtained geographical coordinates of each cattle farms from the Danish Central Husbandry Register (CHR) [31].

### Estimation of microclimatic temperatures at Danish cattle farms

We obtained meteorological data from the implementation of the numerical weather prediction model system HIRLAM (High-Resolution Limited Area Model) at the Danish Meteorological Institute (DMI). Details of the dynamical and numerical aspects of the model can be found in the HIRLAM Scientific Documentation [32], and the DMI implementation is described by Sass et al. [33]. The meteorological data, dating back to the year 2000, are available in a circumpolar horizontal grid. The grid covers Europe and large parts of northern Asia and the Atlantic at a spatial resolution of approximately 15 km, and has an hourly time resolution. At the synoptic times 0, 6, 12 and 18 UTC (Coordinated Universal Time), the model assimilates a large number of the various different meteorological observations available in the geographical domain. The model calculates the initial state for the model integration. This analyzed state is a solution to governing equations of the atmosphere, as implemented in the model, in accordance with the observational data [26]. The model predictions at the intermediate synoptic hours (1–5 UTC, 7–12 UTC, and 19–24 UTC) are used.

In this study, we used the model temperature at a height of 2 m above the ground. We obtained the hourly meteorological temperatures, solar radiation, wind speed and humidity for each cattle farm according to the nearest model grid point. We then quantified the area of each of the different land covers within a radius of 500 m of each cattle farm in Denmark *(n* = 22,092) using the CORINE Land Cover database, 2006 [34]. We used CORINE Land Cover level 3 to classify the land cover, as it provided the highest resolution of vegetation information [34]. In total, 49 different types of land cover are described in the CORINE database, 25 of which we assumed to be suitable vector habitats. We did not have suitable microclimatic models for four of the 25 CORINE land covers (beaches, dunes, sands, bare rocks, and burnt areas). We reclassified the remaining 21 land covers into four major habitats: (i) dry meadow (non-irrigated arable land, rice field, pasture, permanent crops, complex cultivation patterns, natural vegetation, natural grasslands, moors and heathland, sparsely vegetated area); (ii) hedges (fruit trees and berry plantations, transitional woodland-shrub, vineyards, olive groves); (iii) wet meadow (permanently irrigated land, inland marshes, intertidal flats, estuaries); and (iv) forest (agro-forestry areas, broad-leaved forest, coniferous forests, mixed forest, sclerophyllous vegetation). These four microhabitats are described by Haider et al. [10]. We then regrouped the CORINE land cover surrounding the cattle farms into these four major microhabitat types, omitting land cover types that could not be reclassified into these microhabitat types. We estimated the hourly microclimatic temperatures at three different heights, using recently published microclimatic temperature prediction models for dry meadow, wet meadow, hedges, and forest [10]. In this study, we considered the temperature at 0.55 m above ground for dry meadow, 2.2 m above ground for hedges, 6.8 m above ground for forest, and 0.50 m above ground for wet meadow, based on a literature review [15, 22, 35], expert opinion, and our assumption that these heights were representative of *Culicoides* spp. resting sites.

### The microclimatic temperature prediction model

The microclimatic model uses hourly standard meteorological recordings as input variables to predict the hourly microclimatic temperature of a particular habitat [10].

Microclimatic temperature = meteorological temperature + meteorological temperature the previous hour + solar radiation + wind speed + humidity + month weight (from May to October) + time of day + (solar radiation * wind speed) + (solar radiation * month) + (wind speed * height above ground) + (solar radiation * height above ground)

The weight of months was calculated with the formula

$$ \mathrm{Month}\ \left(\mathrm{x}\right)=15\hbox{--} \left(\mathrm{absolute}\ \left(\mathrm{date}\right)/\mathrm{No}.\mathrm{of}\ \mathrm{days}\ \mathrm{of}\ \mathrm{month}\right) $$

The fitted model was used to predict hourly microclimatic temperature for each of the Danish cattle farm for the period of 2000–2016.

The microclimatic models were developed for the period May to October [10]. We furthermore estimated the microclimatic temperature for the period April to November with the assumption that the habitats would remain the same in November as for October, and April would be the same as for May.

Since the precise resting sites of *Culicoides* spp. are not known, we assumed that vectors would select a resting habitat randomly and that this would be proportional to the availability of the habitats around the farms. If for example, a farm was surrounded by 60% dry meadow and 40% forest, we assumed the 60% of the vectors would permanently rest in the dry meadow and 40% would permanently rest in the forest. However, vectors may actively select a resting habitat according to a preferred temperature or other criteria. To quantify the potential for disease transmission in case vectors actively select a preferred habitat every hour, we estimated the EIP at both the warmest and the coldest habitats available at each farm. We compared the EIP estimated by the different microclimatic temperatures to the EIP estimated by the DMI-modeled temperature for the nearest grid point to each cattle farm.

### Estimating the EIP of Schmallenberg virus

We estimated the EIP of Schmallenberg virus using the following equation, originally developed for bluetongue virus serotype 9, but widely used for Schmallenberg virus [7]:

$$ 1/\left(0.019\times \left(\mathrm{T}\hbox{--} 13.3\right)\right)\ \mathrm{where}\ \mathrm{T}\ \mathrm{is}\ \mathrm{the}\ \mathrm{hourly}\ \mathrm{temperature}\ \left({}^{{}^{\circ}}\mathrm{C}\right) $$

We developed a rate summation model; in which virus development was calculated hourly and summed up daily until virus development was complete (i.e. reached a value of 1). We used the estimated microclimatic temperature of all four classes of land cover (dry meadow, wet meadow, forest, and hedges) to estimate four different EIP for each farm. Assuming the vectors select resting sites completely randomly, we estimated a weighted average EIP (EIP_rand_) for each farm based on the proportion of the four habitats surrounding each farm. We considered the maximum lifespan of *Culicoides* spp. to be 60 days, and the EIPs are presented as average values of the different microclimates at each farm and as averages of different farms. In the model, an EIP of 60 days on May 1st indicates that virus development would be completed on June 29th (60 days later) for any biting midges that ingested an infected blood meal with Schmallenberg virus on that day. Therefore, we included the temperature data up to November 30th so that we could allow 60 days after the last date of our EIP calculation (September 30th). When the temperatures at one or more habitats at a farm were too low for the EIP to complete in 60 days, it became problematic to calculate an average EIP for that farm. Realistically, these sites did not have a value for EIP, as values greater than the lifespan of *Culicoides* spp. are not plausible. However, omitting these cooler sites from the average EIP of a farm would artificially shorter the average EIP by selectively removing the coolest microclimates from the average. To be able to present average estimates of EIP for a farm, we allocated a value of 61 days to the EIP of habitats where virus development was not possible in 60 days. But when the average EIP of all habitats on a farm reached a value of over 60 days, we concluded that EIP could not be completed at that farm.

Vectors may not select their resting sites randomly but may instead be able to move and select a favorable microclimate every hour. We therefore identified the maximum and minimum hourly temperature among the four habitats surrounding each farm and used these time series to estimate the maximum temperature EIP (EIP_maxT_) and the minimum temperature EIP (EIP_minT_). We also estimated the EIP using DMI’s modeled temperature (EIP_DMI_). We estimated the EIP for each transmission period (April 1st - September 30th) for the years 2000–2016, and finally calculated a 17-year average EIP for each season: spring (April 1st - May 31st), summer (June 1st - August 31st) and autumn (September 1st - September 30th). Finally, to supplement the modeled microclimatic temperatures, we also calculated the EIP using hourly maximum and minimum microclimatic temperature recorded in the field at Strødam, 30 km North of Copenhagen, Denmark, during 2015. The details of the data collection are described in Haider et al. [10]. The EIP model was developed using the statistical software SAS [36]. We used the statistical software R version 3.4.0 (packages “*raster*”, “*maptools*” , “*rgdal*”, “*plyr*”, “*foreign*” “*lubridate*”) to predict the hourly microclimatic temperature for each year in order to perform summary statistics of temperature and EIP data and to produce all figures [37]. All maps were prepared in the geographical software QGIS [38].

## Results

### Land cover

There were 22,092 cattle farms in the CHR database. Of these, 22,004 farms were surrounded by at least one of the four habitats: dry meadow (83% of farm areas), hedges (6%), wet meadow (3%) and forest (3%). The remaining 5% of farm areas were covered by habitats for which we had no model to estimate the microclimatic temperature (e.g. beaches, dunes, sands, etc.). The remaining 88 farms (0.4%) either did not contain any habitats included in the microclimate model, or contained habitats that were not suitable as vector-resting sites. We excluded these farms from further analysis. Of the 22,004 farms, 8448 (38%) had only one of the four types of land cover: 8444 had only dry meadow, three had only hedges and one farm had only forest within a 500 m radius.

### Comparison of microclimatic and DMI-modeled temperatures of Danish cattle farms

**Fig. 1:**
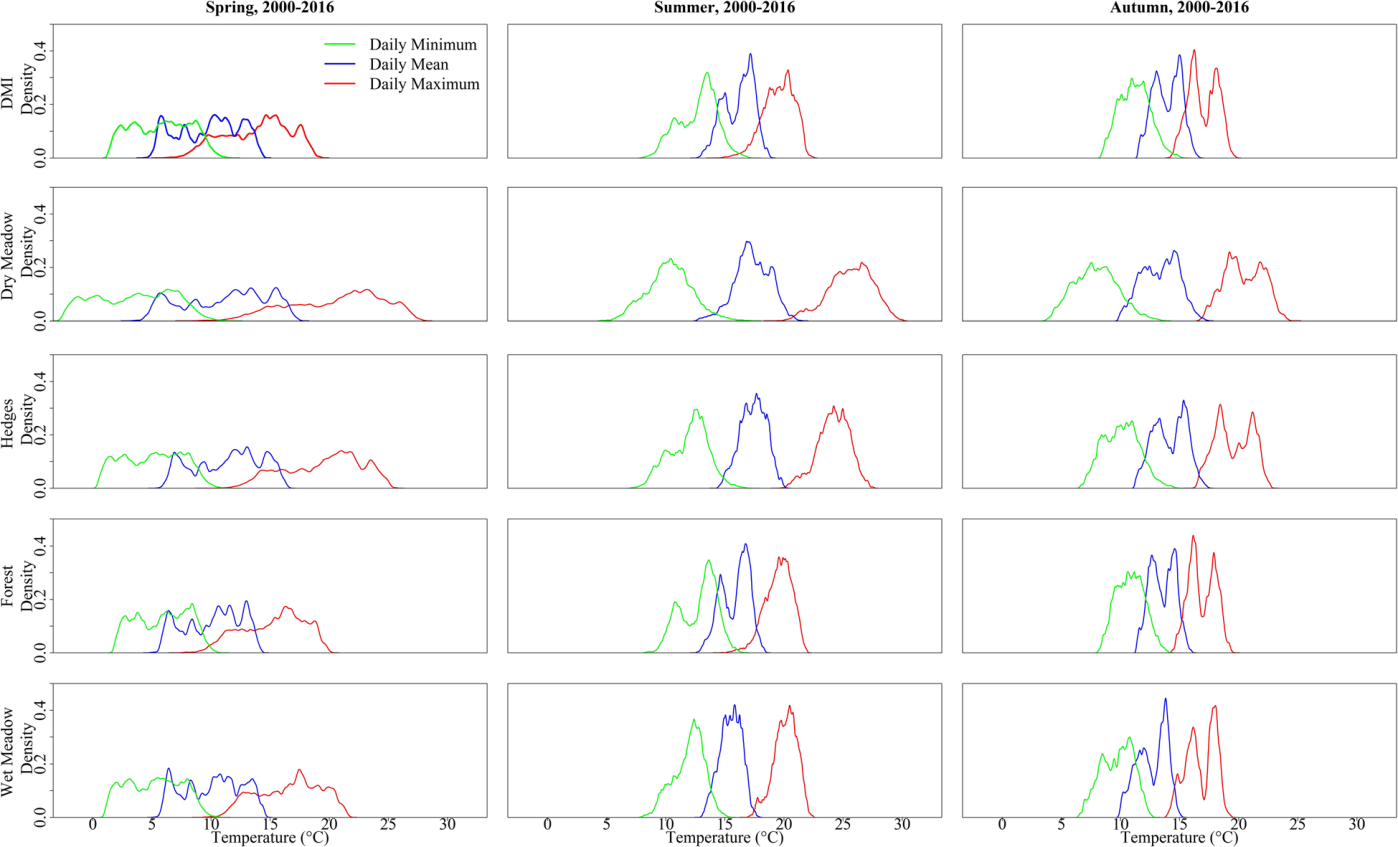
The relative frequency of daily maximum, minimum and mean temperatures for four microclimatic habitats and standard Danish Meteorological Institute (DMI) temperatures surrounding Danish cattle farms (500 m radius for microclimatic habitats), based on hourly temperature data. The figure represents 17-year (2000–2016) averages for spring (April-May), summer (June-August), and autumn (September)

The average daily minimum, maximum and mean temperature of each of the four habitats surrounding the cattle farms for the 17 year-period are summarized in Fig. 1, together with the standard DMI temperature. The estimated microclimatic temperatures differed considerably from the DMI temperature. This difference was larger in spring and autumn than summer. In spring, the daily maximum temperature varied (5th and 95th percentiles) from 9.0 °C to 17.9 °C (DMI-modeled), 13.7 °C to 26.2 °C (dry meadow), 13.9 °C to 24.1 °C (hedges), 12.1 °C to 20.6 °C (wet meadow), and 10.7 °C to 18.9 °C (forest). During the same period, the minimum temperature varied (5th and 95th percentiles) from 2.0 °C to 9.5 °C (DMI-modeled), -1.7 °C to 8.4 °C (dry meadow), 1.1 °C to 8.9 °C (hedges), 1.6 °C to 8.7 °C (wet meadow), and 2.4 °C to 9.1 °C (forest). The dry meadow habitats had the most extreme temperatures, with the warmest daytime temperatures and coldest nighttime temperatures during the period April to September 2000–2016 (Fig. 1). On average, the daily maximum temperature in dry meadow, hedges, wet meadow, and forest was 3.9 °C, 3.1 °C, 0.9 °C and 0.4 °C higher than the DMI daily maximum temperature, respectively. The average daily minimum temperature in dry meadow, hedges, wet meadow, and forest was 3.4 °C, 1.1 °C, 1.1 °C and 0.1 °C lower than the DMI daily minimum temperature, respectively. The DMI estimates for daily maximum temperature of different farms located in different parts of the country varied (5th and 95th percentiles) from 10.4 °C to 21.1 °C (difference: 10.7 °C), and the daily minimum temperature varied (5th and 95th percentiles) from 1.0 °C to 14.3 °C (difference: 13.3 °C). The warmest habitat of a farm had an average temperature that was 3.7 °C (5th and 95th percentiles: 1.0–7.8 °C) higher than the coolest habitats of the same farm.

### Spatial variation of temperature in Denmark

**Fig. 2:**
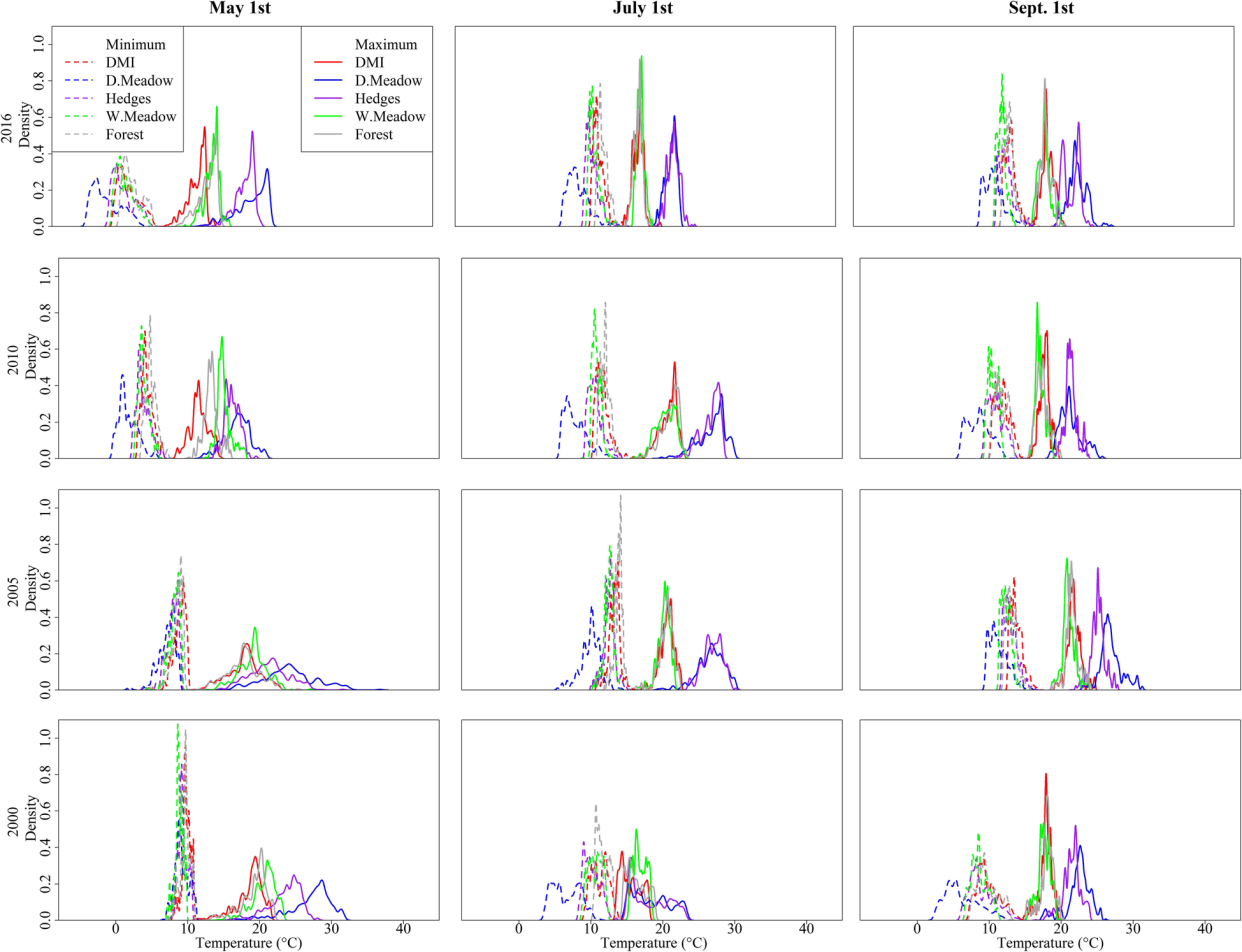
The daily maximum and minimum temperatures of microclimatic habitats within a 500 m radius of Danish cattle farms. The figure represents the daily minimum and maximum temperatures on May 1st, July 1st and September 1st for four selected years: 2000, 2005, 2010 and 2016

To quantify how temperature varied spatially on a particular day, we plotted the minimum and maximum temperature for each farm on May 1st, July 1st and September 1st in four selected years: 2000, 2005, 2010 and 2016 (Fig. 2). This showed a wide variation in daily temperatures in Denmark. For example, on May 1st 2016, the maximum temperature varied (5th and 95th percentiles) from 15.5 °C to 22.2 °C in dry meadow, 16.4 °C to 20.3 °C in hedges, 12.4 °C to 15.5 °C in wet meadow, and 11.1 °C to 15.2 °C in forest habitats, compared to a variation of 9.7 °C to 13.5 °C in DMI-modeled temperatures (Fig. 2). The minimum temperature on the same day varied (5th and 95th percentiles) from -2.9 °C to 3.2 °C in dry meadow, 0.1 °C to 4.8 °C in hedges, 0.6 °C to 4.6 °C in wet meadow, and 1.6 °C to 5.6 °C in forest habitats, compared to 0.8 °C to 5.4 °C in DMI-modeled temperatures (Fig. 2).

### Comparison of the EIP of Schmallenberg virus estimated from different temperatures in Danish cattle farms

**Fig. 3:**
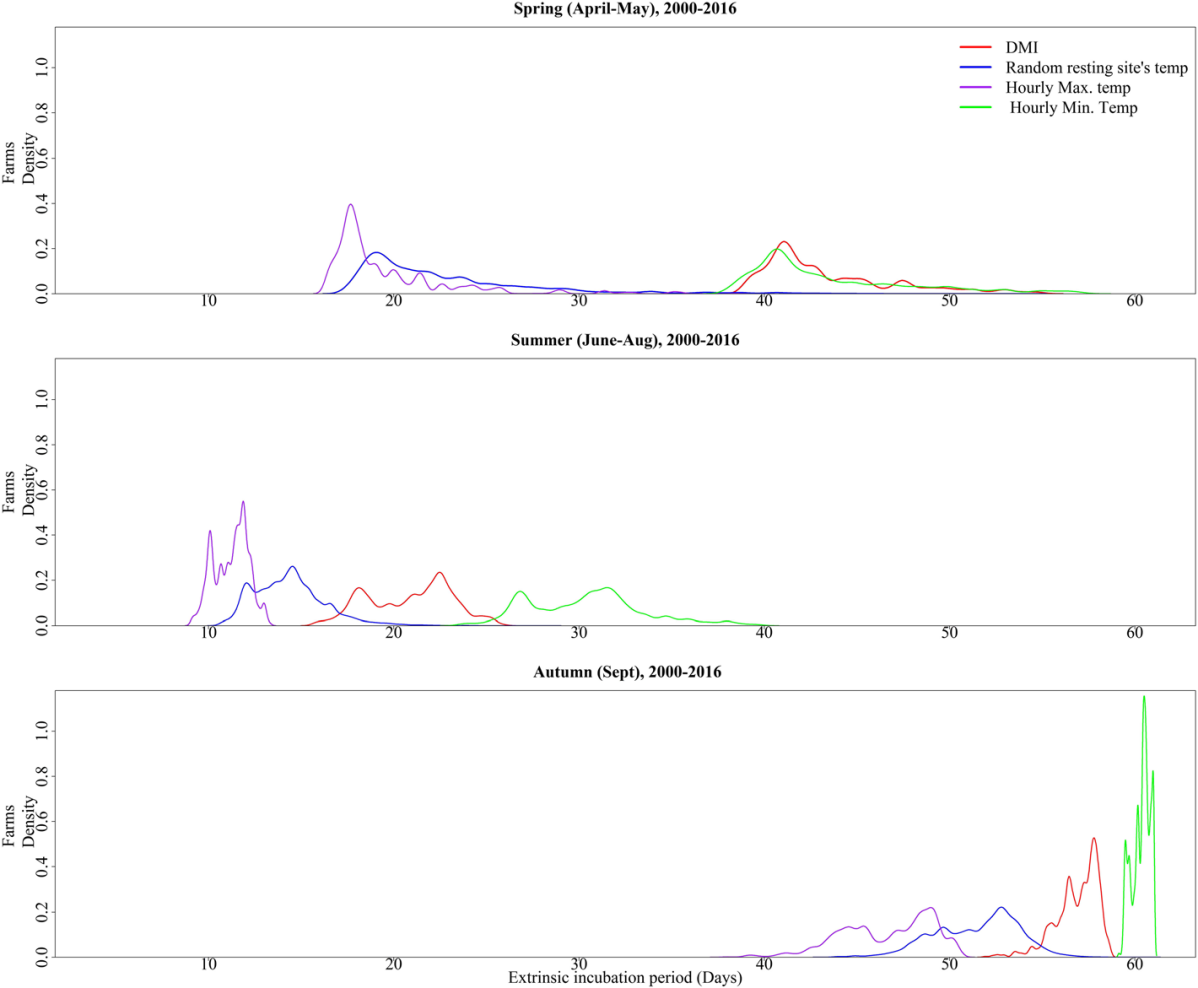
The extrinsic incubation period (EIP) of Schmallenberg virus on Danish cattle farms. The figure illustrates the 17-year average EIP for spring (April 1st - May 31st), summer (June 1st - August 31st), and autumn (September 1st - September 30th), assuming that vectors either randomly select a resting site according to the area this habitat occupies within a 500 m radius of the farms (random resting-site), or the warmest (hourly maximum temperature) or coldest (hourly minimum temperature) available farm habitat each hour, or that they rest at the nearest DMI temperature grid point (DMI)

The mean EIP of Schmallenberg virus (5th and 95th percentiles) for all cattle farms during spring, summer, and autumn for the 17-year period was: 23 (18–33), 14 (12–18) and 51 (48–55) days, respectively, assuming that vectors select resting sites randomly. These estimated EIP values were much shorter than the EIP generated from DMI temperatures, which were: 43 (39–52), 21 (17–24), and 57 (55–58) days, respectively. The EIP of Schmallenberg virus estimated from random resting sites was comparable to the EIP estimated from the hourly maximum temperatures at the farms for the same three periods: 20 (17–26) days (spring), 11 (10–13) days (summer), and 46 (42–50) days (autumn). However, the EIP estimated when vectors were assumed to select the minimum hourly temperatures at the farms were much longer: 44 (39–53) days in spring, 30 (26–36) days in summer, and 59 (59–60) days in autumn (Fig. 3).

### Annual variation in Schmallenberg virus EIP

**Fig. 4:**
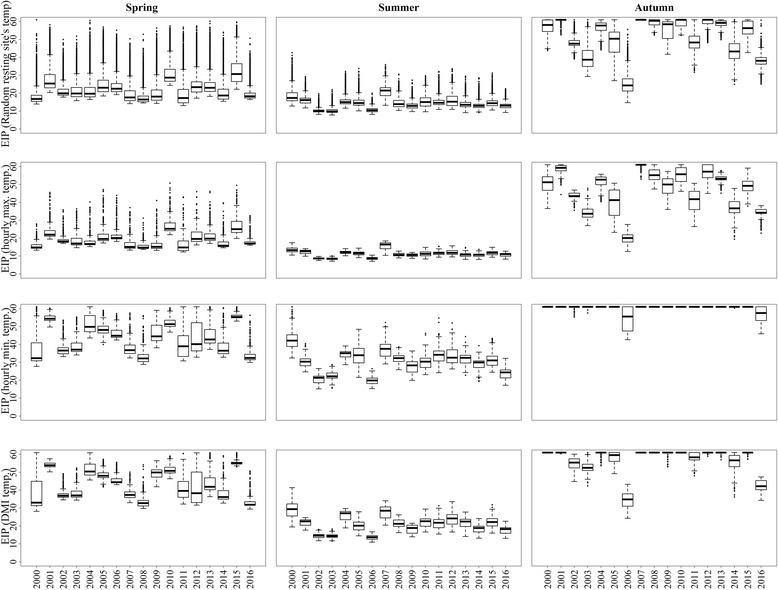
The annual variation in the extrinsic incubation period (EIP) of Schmallenberg virus between 2000 and 2016 at Danish cattle farms in: spring (April-May), summer (June-August), and autumn (September), assuming that vectors randomly select a resting site according to the area this habitat occupies within a 500 m radius of the farms (random resting), or that the vector chooses the warmest (maximum temperature) or the coldest (minimum temperature) available farm habitat every hour or that they rest at the nearest Danish Meteorological Institute (DMI) temperature grid point. The bottom and top of the box indicate the first and third quartiles; the band inside the box is the median. The dots outside the box are outliers

There was a large year-to-year variation in the EIP of Schmallenberg virus over the three seasons for the period 2000–2016 (Fig. 4).The mean EIP of Schmallenberg virus in the spring of two consecutive years, 2015 and 2016, was 31 and 19 days using random resting-site temperatures, 27 and 18 days using hourly maximum temperatures, 56 and 34 days using minimum hourly temperatures, and 55 and 33 days using DMI temperatures (Fig. 4). In general, the EIP of Schmallenberg virus infections starting in the vectors during summer was shortest, followed by infections starting in spring and autumn (Fig. 4). In the spring of 2012, Schmallenberg virus was detected in a malformed calf born in Denmark, when the mean EIP for all Danish cattle farms was 24 and 42 days based on an estimation of random resting-site and DMI temperatures, respectively. The cow was probably infected during the autumn of 2011, when the mean EIP was 48 and 59 days, respectively.

### Spatial variation in EIP

**Fig. 5:**
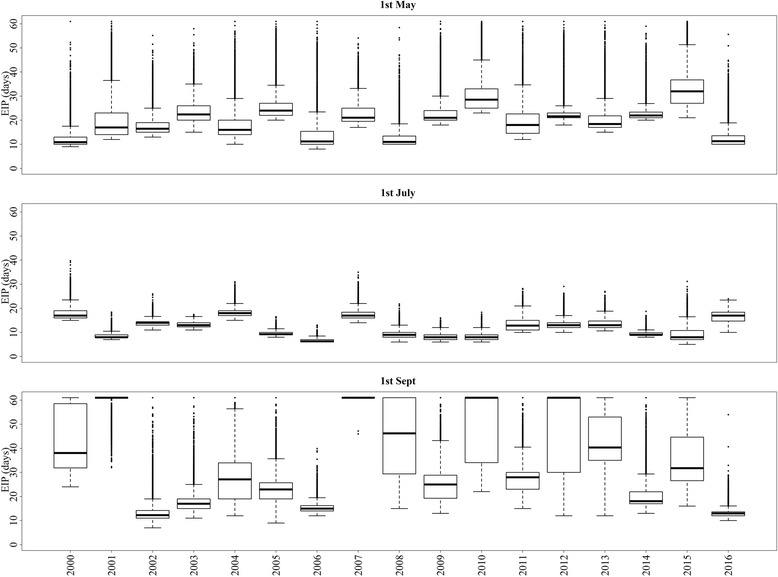
The virus development time (extrinsic incubation period) in *Culicoides* spp. vectors when infected with Schmallenberg virus on Danish cattle farms on May 1st, July 1st and September 1st during the period 2000–2016, assuming that vectors randomly select a resting microclimatic site according to the area this habitat occupies within a 500 m radius of the farms. The bottom and top of the box indicate the first and third quantiles, the band inside the box is the median. The dots outside the box are individual outliers

**Fig. 6:**
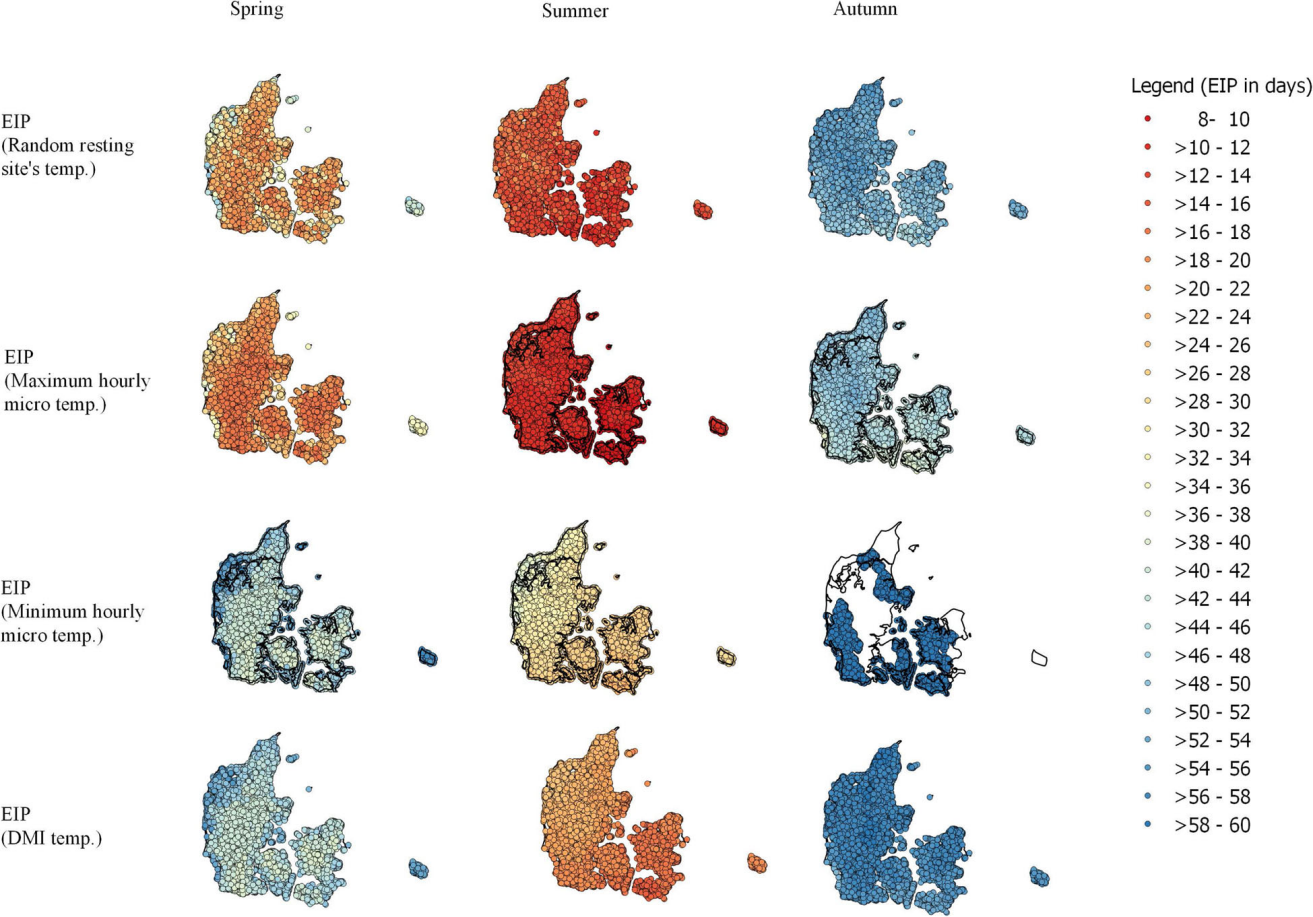
Map of Danish cattle farms showing the 17-year average extrinsic incubation period (EIP) of Schmallenberg virus in spring (April 1st - May 31st), summer (June 1st - August 31st), and autumn (September 1st - September 30th) using random resting-site temperatures, Danish Meteorological Institute-modeled (DMI-modeled) temperatures, and maximum and minimum hourly microclimatic temperatures. The EIP is generated from our virus development model using estimated hourly microclimatic temperatures. The white color indicates the farms where virus development is not possible in 60 days on average

The variation in EIP over a short time period (e.g. 1 day) differs due to the spatial variation of temperatures in farms located in different parts of the country. We plotted the distribution of EIP_rand_ estimated based on random resting sites’ temperature for specific dates (May 1st, July 1st, and September 1st) for each of the 17-year period for all Danish cattle farms in order to examine the geographical variation in Schmallenberg virus transmission potential (Fig. 5). There was a large variation in EIP (5th and 95th percentiles) between farms: 9–19 days on May 1st^,^ 2000, 21–40 days on May 1st^,^ 2005, 23–43 days on May 1st^,^ 2010, 25–56 days on May 1st^,^ 2015, and 10–21 days on May 1st 2016 when modeled with temperatures from random resting sites. For July 1st, the estimates (5th and 95th percentiles of EIP) were: 16–23 days in 2000, 9–11 days in 2005, 6–11 days in 2010, 6–15 days in 2015, and 12–20 days in 2016. For September 1st, the estimates were: 29–60 days in 2000, 11–32 days in 2005, 24–60 days in 2010, 18–60 days in 2015, and 11–16 days in 2016 (Fig. 6). The daily variation in EIP between farms was larger in May and September. A large geographical variation in EIP on a particular day was observed over the 17-year period (Fig. 5).

### Geographical patterns of Schmallenberg virus EIP in Denmark

In general, cattle farms located in the southeastern part of the country (comprising southern Funen and associated islands, Lolland, Falster, and southern Zealand) had a shorter EIP. Farms located in Jutland, especially those in the north-west (comprising Thisted and Herning), had a longer EIP (Fig. 6). This pattern applied to all calculations of the EIP, whether we assumed that vectors were resting at random resting site temperatures, at maximum temperatures, at minimum temperatures or at the DMI temperatures. The maps based on the selection of random resting sites showed that farms with a shorter EIP (in red) were surrounded by a number of farms with a longer EIP (in blue). This indicates that land cover around the farm plays an important role in determining the EIP of Schmallenberg virus, rather than a climatic geographical trend alone. At minimum hourly microclimatic temperatures, the EIP could not be completed in over half of the farms (*n* = 12,030, 54.7%) during the autumn.

### Variation in EIP with different types of temperature

**Fig. 7:**
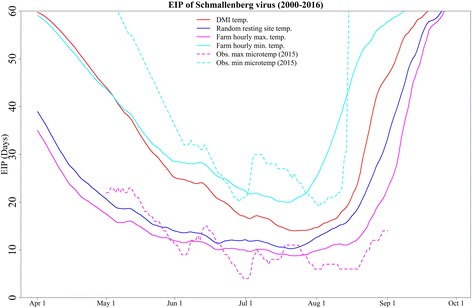
The daily average extrinsic incubation period (EIP) of all cattle farms for the period 2000–2016 (April 1st - September 30th). The estimated EIP using random resting-site temperature (blue line) showed a shorter virus development period compared to the Danish Meteorological Institute (DMI) temperature (red line). Using estimated maximum and minimum hourly temperatures at Danish cattle farms generated two extreme scenarios of EIP (purple and cyan lines). The observed microclimatic temperature from 2015 only showed real annual variation in EIP for Schmallenberg virus for this specific year (dotted line). The observed field data from Strødam were available from May 1st, 2015 to September 1st, 2015

On average, from April 1st to September 30th, the minimum number of days required for completion of the EIP was: 10 days with the random resting-site temperature, 9 days with the hourly maximum temperature, 20 days with the hourly minimum temperature, and 14 days with the DMI temperature (Fig. 7). The range of the EIP for the year 2015 at Strødam was 4–23 days based on the observed maximum microclimatic temperature and 19–60 days based on the observed minimum microclimatic temperature (Fig. 7). When assuming that the vectors selected the lowest available temperature at each farm for each hour for the entire transmission season (April 1st to September 30th), the EIP was on average 2.3 (range: 1.1–4.1) times longer than the EIP for the same farm with vectors assumed to select the maximum microclimatic temperature. The EIP based on random resting-site temperatures was shorter throughout the transmission seasons than the EIP estimates based on DMI temperatures. The EIP based on random resting-site temperatures also showed a longer season of transmission.

### Land cover and EIP (based on random resting sites’ temperature)

The mean EIP for farms with 10% dry meadow varied (5th and 95th percentiles) from 25–33 days, whereas the estimates varied from 21–29 days for the farms with 75% dry meadow. The mean EIP for farms with 10% forest varied (5th and 95th percentiles) from 23–30 days, whereas the estimates varied from 27–35 days for the farms with 75% forest. This suggests that the proportion of warm and cold land cover around the farm had an important role in driving farm-level EIP.

## Discussion

Sixty-three percent of infectious diseases in Europe are climate sensitive and 82% of these are sensitive to temperature alone and vector-borne diseases have been identified as the most temperature-sensitive diseases [39]. Our study generated one of the largest microclimatic temperature datasets in Europe. The dataset is useful for modeling temperature-sensitive diseases of livestock and diseases with zoonotic potential as microclimatic temperatures of Danish cattle farms are different from the temperatures modeled by National Meteorological Institute, DMI. While a single temperature is modeled by DMI for a specific geographical location, we found that approximately 62% farms had more than one type of land cover and therefore more than one microclimatic temperature at a specific point in time. Dry meadow was the most abundant habitat type and had the warmest microclimatic temperature, whereas wet meadow was the coolest and least abundant habitat type in Denmark.

The microclimatic habitats surrounding the farms were 0.4 °C to 3.9 °C warmer or 0.1 °C to 3.4 °C cooler than the DMI-modeled temperature. Daily temperatures observed on farms located in different parts of the country could vary by a maximum of 10.7 °C to 13.3 °C based on standard meteorological office data, but there were microhabitats within a 500 m radius on a farm in which temperatures could vary by a magnitude of 1.0 °C to 7.8 °C (mean: 3.7 °C) each hour. This emphasizes the importance of variation in microclimatic habitat temperature and the need to incorporate it into vector-borne disease-transmission models. Similar conclusions were made from a microclimatic study in Georgia, USA, where researchers concluded that the climatic condition captured by local weather station data did not reflect the microclimatic temperature experienced by the mosquitoes [13]. Another study conducted in rural Argentina showed that microhabitats were generally 5.0–5.6 °C warmer than the ambient temperature [40]. A further study in tropical urban settings in Chennai, India reported higher daily mean temperatures in microhabitats than was recorded by weather stations [14]. A study conducted in the Netherlands showed similar daily mean temperature recorded from national meteorological institute and microclimatic data loggers but the daily temperature variation was much larger in microclimatic habitats [15]. Such variation might have a large impact on virus development and insect survival [15, 41]. The differences in temperature modeled by DMI and the temperature, we predicted for microclimatic habitats, resulted in a large variation in the estimates of EIP which is in an agreement with the Chennai, India study [14]. Here the EIP for both the vivax and falciparum malarial parasites was found to be 1–4 days shorter using measured microclimatic temperature compared to meteorological temperatures [14]

An important finding of this study was the large between-farm variation in the EIP of Schmallenberg virus. Denmark is a small country of 42,931 km^2^, throughout which the mean monthly temperature does not vary more than 2 °C. It has therefore been assumed that vector-borne diseases have only small climate-driven variations in transmission patterns. However, we found a large variation in the EIP for cattle farms located in different parts of the country. The EIP of Schmallenberg virus varies from the coolest to the warmest site on a farm by a factor of 1.1 to 4.1. This means that the virus could develop in the biting midges in seven days in one type of habitat at a cattle farm, and up to 29 days in another habitat at the same farm, despite the midges being infected on the same day.

We found a consistent geographical pattern that showed farms with shorter EIP for Schmallenberg virus were grouped together in the southern parts of the country. Such microclimatic hotspots are important as they may help veterinary authorities prioritize areas for surveillance and allocate resources to prevent and control potential outbreaks, e.g. by increasing vaccination cover locally. This finding has practical implications for Denmark and similar areas in temperate climates around the world. Countries and territories may need to implement strategies to identify, control and prevent vector-borne diseases based on how rapidly a virus can develop within the area, and farm habitats might play a vital role in such decisions. Particular attention may be necessary for parts of a country that is rich in a particular habitat thought to increase the risk of vector-borne disease transmission (e.g. dry meadow). While performing risk assessments for vector-borne diseases, the farm-level potential for disease transmission (e.g. EIP) should be assessed thoroughly, together with other important transmission parameters that can vary spatially, e.g. vector abundance and host densities.

There is an increased concern that climate change might affect the transmission of vector-borne disease in terms of greater geographical expansion of existing diseases and a higher number of outbreaks in endemic areas[42] as climate change may increase the reproductive rate of the insect, the insect biting rates, and shorten the pathogen incubation period [43]. The Intergovernmental Panel on Climate Change (IPCC) have projected a rise in temperature of *c*.0.2 °C per decade over the next two decades [44]. In our study, we found that *Culicoides* spp. have available microhabitats surrounding a farm that are on average 3.7 °C warmer than the coolest habitats. If the biting midges can choose habitats optimally, the variation in the microclimatic temperatures they can be exposed to is much larger than what is predicted due to global warming over the next two decades. It has been suggested that man-made changes in highland Africa have caused an increase in microclimatic temperatures, thereby increasing the vector abundance and facilitating malaria transmission [45]. Climate change could worsen the condition in future [42, 43], but the impact can potentially be counteracted by a change in vector-resting behavior, or by a change in land cover. This has been shown in studies in Austria, where land cover classes were reported to be the most significant factor for the abundance and distribution of mosquitoes [46]. In Uganda, replacement of natural swamp vegetation with agricultural crops led to increasing temperatures, contributing to higher malaria transmission [45]. A change in resting sites could lower the resting temperature of vectors even if the global temperature increases. Such a change may have a bigger impact than years of global warming. Mordecai et al. [47] showed that a 6 °C temperature decrease in the optimum temperature for malarial pathogen development is equivalent to a century of temperature change projected by worse-case climate change scenarios. Therefore, there may not be a simple relationship between global warming or increasing temperatures and vector-borne disease transmission. Instead, the impact is complicated and highly dependent on the microhabitat of the resting sites as well as the vector-resting behavior as shown in earlier studies of malaria in East Africa [41]. Here they evaluated the malaria parasite development rate at different temperatures and found that mosquitoes resting indoors at warmer temperatures could transmit malaria between 0.3 and 22.5 days earlier than mosquitoes resting at colder outdoor temperatures [41].

We found a wide range of EIP for three different estimates of microclimatic temperatures and the standard DMI temperatures. For example, the minimum number of days required to complete the EIP using the warmest hourly temperature for a farm over the entire transmission season (April-September) was 9 days, whereas the estimate was 20 days for the coolest hourly microclimatic temperature. Therefore, the choice of input temperature has a very large impact on the model outcome, stressing the importance of selecting appropriate temperatures for modeling vector-borne diseases. Standard meteorological temperatures are often used for modeling vector-borne disease, yet it is not reasonable to assume that these will represent an average of the actual resting-site temperatures.

Although we do not know the precise location of vector-resting sites, a number of studies have looked at the resting sites of biting midges [15, 20–22, 25, 26]. These studies showed that biting midges can choose habitats between a few centimeters and 10 m above ground, and can choose favorable microclimatic habitats from up to 1.75 km [15, 21, 27]. It has recently been shown that land cover type could significantly affect the distribution of mosquitoes [46]. They might select shaded and humid places during the warm hours of the day, and warmer areas during cooler periods of the day/night. Humidity, shade, and temperature may all play an important role in resting-site selection, but the temperature will ultimately affect virus development. This again emphasizes the need for a better understanding of insects’ selection of resting sites, to identify the appropriate temperatures for modeling vector-borne diseases.

Our estimates of EIP support the empirical findings in Denmark and other European countries [14, 17, 48]. On September 30th, the mean EIP of Schmallenberg virus was 21 days using hourly maximum temperatures, 35 days using random resting-site temperatures, 49 days using DMI temperatures and 60 days using hourly minimum temperatures. This shows that even at minimum microclimatic temperatures, biting midges will be able to transmit the virus 60 days later (i.e. in the last week of November). Pregnant ewes and cows infected in mid to late November will give birth to malformed lambs/calves around March-April and August-September the following year. Schmallenberg virus has recently been identified in aborted sheep/cattle during spring in Belgium [48] and in Denmark [4]. We found a relatively long season of transmission when modeled with microclimatic temperature compared to that of DMI temperature. We considered the maximum lifespan of biting midges to be 60 days. In reality, the survival of biting midges depends on many factors and the lifespan of *Culicoides* spp. has been documented to vary widely, from 10 to 90 days [49]. We estimated the EIP of Schmallenberg virus for the period of April 1st to September 30th, deeming this to be warm enough to facilitate vector-borne disease transmission.

In an extreme scenario, we found four days to be the minimum required time to complete the EIP using the observed (maximum) microclimatic temperature recorded at Strødam, Denmark. This indicates that virus development could take just over half a week, even in Scandinavian climates.

The EIP estimated using random resting-site temperatures was very similar to the EIP estimated using the hourly maximum temperature at a farm. This is because the dry meadow was the dominant microclimate (83%) in Danish cattle farms, and this type of habitat being the warmest microclimate among the four habitats included in this study. Therefore, the average farm-level EIP of random resting sites was highly influenced by the dry meadow temperature. The EIP estimated from DMI was consistently longer than the estimates derived from the hourly maximum temperature at a farm, and even the estimates derived from the temperature of random resting sites. Thus, modeling with DMI temperatures will lead to an underestimation of the real potential of vector-borne diseases.

## Conclusions

We estimated a wide range of the EIP of Schmallenberg virus from different microclimatic and DMI temperatures, which highlights the importance of selecting appropriate temperatures for modeling vector-borne diseases. At any given time, the EIP could vary more than fourfold between the coolest and the warmest microclimates of a cattle farm. This finding has important implications for Denmark and other temperate areas around the world, as countries may need to implement strategies for the control and prevention of vector-borne diseases based on the potential for transmission in different geographical areas. The between-farm variation in EIP is large, with a geographical trend suggesting that disease transmission may vary substantially among regions, even in a small country like Denmark. This could be useful when designing risk-based surveillance for emerging and re-emerging vector-borne diseases. To maximize the use of the available resources, surveillance may focus on geographical areas most at risk (for example, farms surrounded by dry meadow) and on high-risk periods (for example July and August), while also taking into consideration other important factors including vector abundance and host densities. About two thirds of cattle farms (62%) in Denmark had more than one type of land cover and therefore more than one microclimatic temperature. We have shown that warmer microhabitats available to *Culicoides* spp. around farms had on average 3.7 °C higher temperatures compared to the cooler available habitats of the same farm. Man-made changes to the habitats surrounding the farms could alter the risk of vector-borne disease transmission in the future. The completion of virus development (and thereby the transmission potential for vector-borne diseases) will be determined by the temperatures of the actual microclimatic habitats in which the vectors rest. This emphasizes the need for better knowledge on the behavior behind insect resting-site selection to enable selection of appropriate temperatures for modeling vector-borne disease transmission. The farm-level microclimatic hourly temperature dataset generated in this study is one of the largest (22,004 farms, each for 8 months for 17 years) used for studying infectious and vector-borne diseases driven by temperature. In the absence of known resting sites, we recommend using the range of possible microclimatic temperatures available.

## Abbreviations

CHR: Central husbandry register
CORINE: Coordination of information on the environment
DMI: Danish Meteorological Institute
EIP: Extrinsic incubation period
HIRLAM: High-resolution limited area model
QGIS: Quantum global information system
SAS: Statistical analysis software
UTC: Coordinated universal time
